# Child food insecurity in the wake of the COVID-19 pandemic: urgent need for policy evaluation and reform in Israel’s school feeding programs

**DOI:** 10.1186/s13584-022-00523-y

**Published:** 2022-02-15

**Authors:** Janetta Azarieva, Elliot M. Berry, Aron M. Troen

**Affiliations:** 1 The Nutrition and Brain Health Laboratory, The Institute of Biochemistry Food Science and Nutrition, The Robert H. Smith Faculty of Agriculture Food and Environment, The Hebrew University of Jerusalem, 76100 Rehovot, Israel; 2 The Hebrew University-Hadassah Braun School of Public Health and Community Medicine, The Faculty of Medicine, The Hebrew University of Jerusalem, 9112102 Jerusalem, Israel

**Keywords:** Food insecurity, School feeding programs, Nutrition policy, Malnutrition, COVID-19, Israel

## Abstract

Even in high-income countries like Israel, children have been particularly vulnerable to the surge in food insecurity driven by quarantines, unemployment, and economic hardships of the COVID-19 pandemic. Under normal circumstances, School Feeding Programs (SFPs) can help to ensure child food security. In the wake of the pandemic, policy makers worldwide have been challenged to adapt national SFPs to provide nutritional support to children (and indirectly to their families) during extended school closures. Most national SFPs implemented contingency plans to ensure continued nutritional support for children. In Israel, where SFPs were largely suspended during long periods of mandated school closing, there was a loss of 30–50% of feeding days for the ~ 454,000 children enrolled in the program. The lack of emergency contingency planning and failure to maintain Israeli SFPs during school closures reveals longstanding structural policy flaws that hindered coordination between relevant ministries and authorities and impeded the mobilization of funds and existing programs to meet the emergent need. The school feeding law does not identify child food security as an explicit aim, there are no benchmarks for monitoring and evaluating the program to ensure that the food aid reaches the children most in need, even routinely, and the Ministry of Education had no obligation to maintain the program and to marshal data on the participants that could be acted upon in the emergency. Moreover, because Israeli SFPs are “selective”, in other words, implemented according to community risk (low-income, high poverty rate) and geographical factors, attendant stigma and financial burdens can make participation in the program less attractive to families and communities that need them the most. We argue that Israel should make urgent, long-term improvements to the SFPs as follows: First, eliminating childhood food insecurity should be made an explicit goal of legislation in the broader context of national social, health, and nutritional goals, and this includes ensuring SFPs are maintained during emergencies. Second, the government should assume responsibility for the routine assessment and data collection on food insecurity among Israeli children. Third, SFPs should be subjected to rigorous independent program evaluation. Finally, a “universal” SFP providing nutritious diets would likely improve the health of all Israeli children, across all socioeconomic backgrounds. These steps to guarantee that Israeli children have food to realize their full physical and cognitive potential would emphasize Israel’s firm commitment to support multiple dimensions of health, educational achievement, and societal values, to combat the complex and long-term consequences of the pandemic, and to prepare for the next one.

## Background

“One cannot equate a person who has bread in his basket to one who hasn’t.”

(Talmud Bavli, Yoma 74b)

### The COVID-19 “stress test”

The COVID-19 pandemic has exposed deeply entrenched social, economic, and health disparities around the world [1–3]. Surging food insecurity is a particularly urgent concern, even in high-income countries, and Israel is no exception. Disruptions to the supply and demand for food, price hikes, widespread unemployment, quarantines and closures, have all restricted physical and economic access to food. Children are particularly vulnerable to food insecurity, which may cause irreversible damage to their physical, cognitive, and emotional development.

The prevalence of child food insecurity in Israel has always been a concern. It was first detailed in the seminal national food security survey by the Myers JDC Brookdale Institute that documented that 22% of Israeli households were food insecure [4]. Follow up studies by Israel’s National Insurance Institute and Central Bureau of Statistics, most recently from 2016, estimated that 638,000, or a quarter of all Israeli children lived in food insecure households and fourteen percent, or 352,000, experienced severe food insecurity. In total, 50.6% of Arab children, 26.3% of Jewish haredi (ultraorthodox) children, and 17.0% of Jewish non-haredi children were food insecure [5]. These surveys measure food insecurity using the Household Food Security Survey questionnaire developed by the United States Department of Agriculture (USDA), which defines food insecurity as “limited or uncertain availability of nutritionally adequate and safe foods or limited or uncertain ability to acquire acceptable foods in socially acceptable ways.” Severe food insecurity describes households that worry seriously about ability to afford and obtain food, and that have significantly reduced the quality or quantity of food they consume, including the experience of hunger.

Childhood food insecurity in Israel is associated with poor metabolic health and paradoxically with obesity as it is elsewhere around the world. Financial constraints and uninformed food choices in food insecure households often lead to poor diets characterized by over consumption of cheap processed and ultra-processed foods and reduced intake of more expensive nutritious food, together with poor lifestyle and exercise habits [6, 7]. As a result, food insecure children may suffer the double jeopardy of excessive caloric intake and micronutrient deficiencies (so-called “hidden hunger”), resulting in a high prevalence of nutritional deficiencies and obesity [8, 9]. The 2015–2016 Israeli National Health and Nutrition (MABAT) youth survey found that 29.9% of middle and high school students were overweight or obese [10, 11]. Current data from the Ministry of Health indicate that in 2021 overweight and obesity remain highly prevalent among Israeli children in elementary school, with rates that increase from 19.5% and 21.9% in Jewish and Arab children in the first grade, to 30.0% and 44.0% in Jewish and Arab children in the 7th grade, respectively (MOH Nutrition Division, personal communication), Thus, even before the COVID-19 pandemic, there was a demonstrable need for a focused and integrated policy response to combat child food insecurity and obesity.

As in other countries, the pandemic has exacerbated this social crisis [12]. It is increasingly urgent to help the growing numbers of economically and newly challenged sectors in Israel’s population that are experiencing food insecurity, a key indicator, and result of poverty. In the absence of current official data [5], “Latet”, a major Israeli food bank, conducted a survey between September and October 2020. Using the USDA questionnaire, they estimated that the prevalence of food insecurity increased significantly during the pandemic to some 656,000 households, or 22.6% of the population. These households included 799,000 children (32%); and for 395,000 children, or 15.8%, the condition was severe [13].

Current national school feeding programs (SFPs) were established in 2005 and are an important policy instrument to provide nutritional support to some the most disadvantaged Israeli children. According to a recent report by the Knesset Research and Information Department, some 454,400 children or approximately 19% of Israeli schoolchildren are currently eligible for the program. However, while school feeding is preferentially implemented in municipalities that are of lower socioeconomic strata and therefore more likely to include children from food insecure households, enrollment is not directly linked to food insecurity, so the programs do not reach all those in need. Moreover, since the SFPs were suspended in almost all schools during several extended school closures, participants lost 30–50% of their school meals over a year [14]. Thus, it is crucial to recognize the negative impact of suspending the SFPs, especially given the widespread economic hardship caused by the pandemic, and develop policies to ensure that they meet the critical and growing needs of food insecure children and their families.

To the best of our knowledge, the current Israeli SFPs have neither been described nor evaluated in the peer-reviewed literature. This narrative review outlines the scope of the existing programs and policy based on government documents and contemporary reports, and provides an initial account of their operation during the pandemic. We hope these data will stimulate discourse, research, and evaluation leading to improvement and reform of the current SFPs.

## Main text

### School feeding programs in Israel before and during the COVID-19 pandemic

SFPs were established in Israel in the decade following independence and terminated in the late 1970s in an era of increased social and economic prosperity [15]. The Knesset re-established them in 2005 and legislated the Daily Meal Law, mandating that pupils attending elementary school until 4 pm, 4 days a week in "extended school day programs" would receive one hot meal per day (lunch). Children with shorter school days are not eligible nor are middle and high school students. It is noteworthy that the law does not mention the social motivation behind the legislation—mitigating food insecurity among children—nor does it explicitly state the social rationale for the extended school day program [16]. The important point here is that the policy should be fully implemented and achieve its underlying purpose.

By law, the ministers of Education and Finance determine which, and how many of the children in extended school day programs are eligible for the SFP. The Ministry of Education, local authorities, and parents are the three main contributors to fund and operate the programs. The cost is shared between the Ministry of Education and the municipalities with the split ranging from 70% of the budget covered by the municipality in communities of high socioeconomic status (7th and 8th deciles) to 20% of the budget in communities from the lowest socioeconomic strata (the 1st decile). Accordingly, municipal authorities are allowed to charge families a percent of the meal cost based on a progressive price schedule tied to the household’s per capita income that is approved by the Knesset Education Committee. The average daily meal cost per pupil is about NIS 15 (~ 4.5 USD) of which parents pay up to 7 NIS. However, due to barriers to means testing, municipal authorities usually set a flat rate for all families; while, at the same time, designated committees provide a discount when appropriate [14, 16].

Using the same method, meals are now provided to students in two additional supplemental education programs, “Milat” and “Nitzanim”, which support children from low-income families in Israel’s periphery. According to the Knesset Research and Information Department, in 2019 “Milat”, provided meals for ~ 23,000 at-risk children, and “Nitzanim” provided meals for 240,000 preschool and first and second grade children enrolled in after school supplemental education. In addition, 191,000 children were eligible for a hot lunch under the original 2005 law because they participate in an extended school day. In other words, SFPs serve a total of ~ 454,000 students [14]. Approximately 45% of these children live in municipalities in the two lowest socioeconomic deciles and 90% in communities below the median. All three programs are run through the Ministry of Education and managed by the Division of Supplementary Learning Programs. In addition to these programs, the Ministry of Labor and Welfare provides meals to approximately 120,000 children in various pre-schools, after-school day care, and boarding schools for at risk youth, but the extent of overlap between children receiving meals in both the Ministry of Welfare and Education programs is unknown.

The SFPs are centrally administered by the Ministry of Education and they operate according to the MOE Director General’s directive (“*xozer mankal*”) which describes the roles and responsibilities of the ministry, municipalities, school principals and food suppliers [17]. The MOE issues a tender for the SFP management company, which is responsible for overall operations, and which currently oversees 25 approved sub-contracted food suppliers. The ministry tender determines the scope of activity of the subcontracted suppliers, and specifies which subcontractor will supply food to the different municipalities and schools. The tender also specifies criteria for the menu according to Ministry of Health dietary guidelines. The SFP management company oversees the food suppliers and monitors food safety, quality and waste [18]. The municipalities or school operators decide on the mechanism of collecting parental payments, e.g. whether these are made to the municipality, the school operators or directly to the selected food supplier. The number of participating children determines the number of meals delivered by the supplier to the school. Meals are delivered either in bulk or individual meal trays [17].

According to a report by the Arlozorov forum, the total Government budget for SFPs in 2019 was 928 million NIS (290M USD) including both Ministry of Education and Welfare budgets, of which some 82%—760 million NIS (81%) was utilized [19]. According to the Knesset Research and Information Department report, the total budget reported for the three programs by the Ministry of Education was 2.15 billion NIS and was comprised of 650 million from the government and 1.5 billion from the municipalities and parent payments [14]. This makes the SFPs the second largest item in the Ministry of Education budget after payroll. Nevertheless, the law does not specify benchmarks for monitoring and evaluating the funding framework to ensure that the food aid reaches the children most in need [16, 20]. Consequently, it is not the children’s needs but rather the municipal authorities’ financial priorities that primarily determine if and how SFPs are locally implemented [21, 22].

In 2009, 4 years after the program was launched, the Israel State Comptroller's Office issued its first critique of the SFPs. The report officially recognized that the primary objective of the School Feeding Program was to alleviate food insecurity among children. It criticized the programs for falling short of this objective, reporting that between 2004 and 2008 the number of eligible pupils *increased* while the number of pupils *actually participating* in the SFPs *decreased* and the fact that the program impacts had not been formally evaluated [20]. Two subsequent State Comptroller reports document continued challenges meeting the SFPs objectives in the context of municipal government programs and the central government’s overall responsibility for combatting food insecurity. Since then, parliamentary oversight committees, the Knesset Research and Information Department, and the National Nutritional Security Council have also documented the on-going challenges of extending enrollment to all food insecure children [14, 23, 24].

A problem in reaching the target populations may stem from the fact that the Israeli SFP takes a “selective” approach. In other words, it is structured according to risk (preferential inclusion of low-income, high poverty rate communities) and geographical factors. The attendant stigma may make participation in the program less attractive to individuals and communities, and needy populations, especially in the periphery, who may be less likely to enrol, as is the case in other countries [25–28]. The relevance of these recognized barriers to participation elsewhere, needs to be evaluated with respect to the Israeli SFPs. Furthermore, because the selective eligibility criteria do not include food insecurity, the schools and Ministry of Education have no data on participants’ food security status, and the programs do not cover food insecure children in middle school and high school.

Clearly, based on the above-mentioned prevalence of child food insecurity, the SFPs do not reach all food insecure children. Indeed, the data from the aforementioned food security surveys indicate that food insecurity reaches into the middle class, and conversely, that not all households and children in Israel’s periphery or lower socioeconomic communities are food insecure. In addition, since meals are provided only when schools are open, many children go hungry over long periods when they are shut down, whether for holidays or due to strikes, and now during long quarantine closures. Moreover, the tri-partite funding structure of the SFPs is even less effective during the present economic crisis, when the Ministry of Education is unable to implement, or be accountable for, the programs on its own, and municipalities and parents are hard pressed to contribute [21, 22]. Finally, the logistic framework, whereby the meals are prepared and delivered by multiple independent and uncoordinated producers who lack any common infrastructure, makes the management of meal production, its quality, and supply inefficient, and a serious impediment to locating and serving those in gravest need, whether during the pandemic or any other national emergency. A “universal” SFP, not predicated on prior criteria such as assessments of food insecurity, might aim to tackle a wider range of societal problems and ensure that all children have access to nutritious food in schools, thereby promoting the health of all children [29, 30].

A recent policy perspective in the New England Journal of Medicine highlighted the crucial importance of ensuring continuity in nutritional support for food insecure children when school is closed, even in high-income countries like the USA, and of reforming SFP policy after the pandemic to this end [31]. Unlike many other countries—such as the UK, USA, Brazil, and India [32], Israel has no contingency plans to provide meals for eligible children during unforeseen school closures. During the first lockdown in March 2020, schools were closed for ~ 30 days resulting in the suspension of all SFPs. Recognizing the probable consequent harm, the Ministries of Education, Health, and Labor, Social Affairs and Social Services appealed to the Finance Ministry and obtained budgetary approval to distribute meals to 18,000, or a mere 4% of children participating in SFPs in the most disadvantaged communities and schools. An additional 17,000 institutionalized at-risk children, or 14% of the ~ 120,000 children in Ministry of Welfare frameworks [14, 33] continued to be fed. The distribution was carried out by the IDF Home front command with the help of community volunteers. Two hearings of the Knesset’s Special Committee for the Rights of the Child in October and November 2020 investigated how and why the Government did not ensure that children would continue to receive such vital food aid during subsequent emergency closures [34, 35]. This lapse was especially egregious during the second closure when the need was known, the budget available, and successful experience on the record from the first closure in the spring. The representative of the Ministry of Education testified that responsibility for the SFPs had been transferred to the Israel Defence Forces’ Home Front Command. However, none of the government officials who testified clarified how this decision had been reached, nor could they provide data on the number of children who had received such meals during the second closure, which lasted between 12 and 37 days depending on age [14, 34, 35]. On January 6, 2021, the government announced a third school closure, which lasted for 25–33 days, again without providing food assistance to children in need [14]. In total, depending on age group, children lost between a third and a half of their school meals during the year from March 2020–2021. This does not include meals lost due to splitting of classes between capsules or children quarantined at home after exposure to ill classmates or teachers, even when schools were officially open.

This brief account highlights the lack of an organized government policy to manage and prioritize programs to address child food insecurity. The failure is twofold: the demonstrable lack of coordination between Ministries and authorities and their inability to mobilize funds and implement existing programs to meet the urgent need. Moreover, the failure to collect official information on the identity and number of children with food insecurity severely hampered efficient delivery of food aid by the relevant authorities during the crisis [34, 35].

### International perspectives on protecting SFPs and child nutrition during the pandemic

National SFPs have been used as a widespread policy tool to achieve a variety of social aims: providing children with a healthful diet to allay malnutrition, fight childhood overweight and obesity, and ensure their full physical, intellectual, and emotional development. School lunches can provide children with nutritional quality superior to food obtained from other sources, even in high-income countries like the USA and the UK, where this is a critically important tool to both narrow social and health inequalities between children from low- and high-income families and to improve dietary quality and lifestyle habits of children across the board [36–39]. Globally, some 368 million schoolchildren in 169 countries participate in SFPs. In high- and middle-income countries, SFPs are almost exclusively financed by the state, while in low-income countries they rely on donor and NGO support that covers up to 83 percent of their costs. Research elsewhere shows the positive impact of SFPs on raising enrollment and educational performance, reducing absenteeism and classroom hunger, and promoting social equity [30, 40]. In middle and high-income countries, SFPs have proven effective both as a welfare policy for alleviating socioeconomic disparities and as a way to inculcate healthy lifestyle habits and to fight obesity in a dignified, non-judgmental, and equitable way [41, 42].

The need to protect child nutrition programs during unforeseen school closures is well described in the nutrition policy literature [43–47]. Many countries and international NGOs such as WHO and UNICEF have contingency plans to feed children when schools are forced to close because of infectious disease outbreaks such as influenza or Ebola, natural disasters, such as hurricanes, earthquakes, tornadoes, and floods, and violent conflicts [48–50]. During the COVID-19 pandemic, school closures in 88 countries affected some 246 million children in SFPs globally. However, the majority of these countries responded rapidly by supporting and strengthening their SFPs so that they could provide alternatives to feeding the children in school during the COVID-19 pandemic closures: 52 countries provided schoolchildren with take-home rations; 14 countries used various other modes of distributing food; while 13 countries provided cash transfers to schoolchildren’s families [32].

Notably, the Indian Government adapted the Mid-day Meals program, (the largest school feeding program in the world), to ensure that schoolchildren continue to receive their basic sustenance during the pandemic. Modalities for distribution range from household cash-based transfers to the delivery of uncooked grains or cooked meals, with decisions left to the states. [32]. In Brazil, the government expanded the Bolsa Familia Program to maintain and even increase coverage including a series of Emergency Cash Withdrawals to support families affected by COVID-19 [32]. In the United States, the Federal Government’s Families First Coronavirus Response Act (March 18, 2020) empowered the US Department of Agriculture to approve state government plans to provide emergency food stamp assistance to households with school-aged children who would otherwise benefit from free or reduced-price meals had there not been school closures and a variety of measures were implemented to coordinate federal and local programs [31, 51, 52]. In the United Kingdom, by the end of March 2020, the government formally launched a national voucher scheme to ensure that the 1.3 million eligible school-aged children continue to have access to meals during school closures. Under the scheme, each school-aged child received weekly vouchers of 15 pounds sterling, redeemable at all major supermarkets. Families with school-aged children could ascertain their eligibility through the government website. The schools were responsible for delivering the vouchers to the relevant pupils [32]. These examples show that it is possible to provide continued food aid to eligible children even during extended school closures, where the importance of feeding vulnerable children is prioritized. However, despite the scope of the problem and significant budget, infrastructure, and expertise, Israel has yet to make this a priority.

### A better way forward

Since the pandemic and its social and economic fallout are still far from over, it is imperative for Israel to develop strategies for the short- and long-term. To provide necessary ongoing food aid to children during emergencies and closures, the government should immediately provide alternative food-assistance, whether through cash vouchers or direct delivery, as described above, notably in India, the US, and Brazil. A designated Ministry or agency should be assigned responsibility for coordinating, implementing, overseeing, and monitoring such emergency responses, and a budget dedicated to ensure continued delivery of food or alternative assistance.

In the long-term, there are three major problems with SFPs that must be addressed with immediate data gathering and planning. First, legislation should be revised to make combatting childhood food insecurity an explicit goal within the broader context of social and health goals, such as academic achievement, nutrition, and lifestyle education and also delineating clear responsibilities and accountabilities. Legislative amendments must codify contingencies to maintain SFPs for future emergency school closures, and regularly monitor the gap between the growing number of food-insecure children and the number of children eligible to participate in government programs. One cannot close the gap if its size is unknown. Contingency plans should support healthy diets for children by allowing for food or cash equivalents to be provided to the families of all participating children through all school grades, and provision should be made to protect food-insecure children during school holidays and the summer months. Moreover, SFPs should be made responsive to changes in the geographical and national distribution of household food insecurity, with mandatory, periodic evaluation, ideally every school year.

Under the existing law, municipal authorities are expected to finance at least 10 percent of the programs’ costs, but they are not challenged when they fail to do so due to budgetary shortfalls, weak administrative capacity, or both. The failure to combat household food insecurity in general, impinges on national security and resilience [53–56]. Ensuring that citizens of the “startup” nation are not living with hunger is unequivocally the government’s obligation and responsibility. Prolonged political indecision has allowed municipal authorities and the Ministry of Education to play “pass the buck” and blame each other for failure to implement the programs. During emergencies, as in the current pandemic, they need to provide timely data to the Ministry of Education, Ministry of Welfare, Ministry of Health, and to the Ministry of Defense and Home Front Command, so they can effectively coordinate and manage emergency food delivery and inform national policy and resourcing [12]. In turn, the central government should provide municipal authorities with adequate funding for these activities, as well as adequate food assistance programs to meet current need [12, 34, 35, 57].

A second requirement, in addition to a change in legislation, is regular surveys to monitor child food insecurity in Israel. The primary source of data at present is the Food Security Surveys conducted by the National Insurance Institute (*Bituach Leumi*) of Israel. The most recent survey in 2016 followed families interviewed in 2011 and 2012 to ascertain the persistence (natural history) of food insecurity. The latest household food security survey conducted by the National Insurance Institute in the first half of 2021 has yet to be published. An additional source is the National Health and Nutrition Surveys (MABAT) carried out by the Ministry of Health,^1^ although these do not specifically focus on food allocation within the household.

Moreover, neither of these cross-sectional surveys focuses on childhood food insecurity, nor are they routine or frequent enough to respond to acute changes in need, such as those experienced during the pandemic and its aftermath. The SFP administration in the Ministry of Education and their interface with municipalities’ schools and social services departments is an obvious channel through which information on the extent of need could be monitored at the level of schools and if necessary individual students. The distribution of meals to 18,000 children during the first closure demonstrates that such coordination is indeed possible.

Legislation is needed to assign responsibility to relevant agencies (e.g. schools, municipal welfare departments, Israel center for disease control) in a cross-cutting collaboration with children at the center, and grant them the necessary authority to collect relevant data, including obesity and malnutrition among children. The lack of such systematic official data and public research makes it difficult to adequately evaluate Israel’s SFPs, to set defined and measurable policy outcomes, and to use the data to improve the program.

More than 15 years after the SFPs inception, a rigorous independent program evaluation is necessary, as is the norm elsewhere [58, 59]. Not until 2015 did the Ministry of Health issue guidelines on children’s nutrition in educational frameworks. The report, "To eat and grow in the afternoon programs" for ages 3–10 years, describes the components of the meal, the frequency of the food items, and portion size [60]. The Ministry of Education which implements the program, carries out the only routine oversight of the SFPs [61]. However, their data, as published online in the “Control of suppliers summary” focus only on food safety and waste [62]. It does not address the social and educational aims of the program, or the problem of obesity, participants’ health, their scholastic and cognitive achievements, emotional wellbeing, and school attendance.

## Conclusions

The current pandemic is a litmus test for the resilience of SFPs in Israel, as elsewhere, and of whether they are adequate to meet their goals as currently designed. It is undeniable that the impact of the COVID-19 pandemic in Israel and its economic and social consequences will long affect the future of society including food security among schoolchildren. Immediate changes and improvements are urgently needed in the short term; and policies must be re-evaluated and adjusted for long-term sustainability. SFPs need to be flexible to accommodate to changing circumstances.

The available data show Israel’s SFPs have inadequately addressed child food insecurity during the pandemic. Participation is not fully realized, and thus despite the considerable public resources invested to achieve the program’s important goals, it has been impossible to assess whether these goals are being achieved. For example, to improve and sustain the SFPs in Israel we need to know: How do the SFPs affect their participants’ nutritional and physical health? How does participation affect their social, emotional, cognitive development and educational performance? How does it affect school enrollment, absenteeism, and atmosphere? How do the SFPs affect families’ budgets and food security? And what is the impact of SFP on the geographical distribution of child food insecurity? This lack of reliable and relevant data of the program’s impact on health and educational outcomes for individuals, their families, and communities, hampers efforts to ensure that the program is effective and adequately resourced.

If neglected, persistent and prevalent food insecurity may have far reaching consequences on the cognitive development of children, and down the line, for national security and resilience. Extensive international experience demonstrates that enforceable government policy can effectively reduce children’s food insecurity. Such a policy will enhance multiple dimensions of health, educational achievement, and society [30, 31, 58, 63]. Israel must make it a priority that all Israeli children have enough nutritious food to achieve their full physical and cognitive potential. Israel can and must use this critical time to develop formal data-driven benchmarks that can be used to adapt, evaluate, and administer SFPs. This can help prepare for the next pandemic and emergency which will surely come [63, 64].

## Data Availability

Not applicable.

## Abbreviations

IDF: Israel Defence Forces
SFP: School feeding program
WHO: World Health Organization

## Footnotes

1 MABAT Surveys are cross sectional national surveys carried out for the Ministry of Health by the Central Bureau of Statistics under the direction of the Israel Center for Disease Control. The surveys include comprehensive self-reported questionnaires on health, demographics and a food frequency questionnaire (FFQ) for different age groups including children. Some iterations have administered a version of the USDA Household Food Security Survey questionnaire, which the National Insurance Institute adapted for use in Israel.
